# The retinal pigment epithelium displays electrical excitability and lateral signal spreading

**DOI:** 10.1186/s12915-023-01559-5

**Published:** 2023-04-17

**Authors:** Irina Ignatova, Roman Frolov, Soile Nymark

**Affiliations:** 1 Faculty of Medicine and Health Technology, Tampere University, Tampere, Finland; 2 Independent Researcher, Oulu, Finland

**Keywords:** RPE, Electrical excitability, Spikes, Ion channels, Patch clamp

## Abstract

**Background:**

The non-neuronal retinal pigment epithelium (RPE) functions in intimate association with retinal photoreceptors, performing a multitude of tasks critical for maintaining retinal homeostasis and collaborating with retinal glial cells to provide metabolic support and ionic buffering. Accordingly, the RPE has recently been shown to display dynamic properties mediated by an array of ion channels usually more characteristic of astrocytes and excitable cells. The recent discovery of canonical voltage-activated Na^+^ channels in the RPE and their importance for phagocytosis of photoreceptor outer segments raises a question about their electrogenic function. Here, we performed a detailed electrophysiological analysis related to the functioning of these channels in human embryonic stem cell (hESC)-derived RPE.

**Results:**

Our studies examining the electrical properties of the hESC-RPE revealed that its membrane mainly displays passive properties in a broad voltage range, with the exception of depolarization-induced spikes caused by voltage-activated Na^+^ current (*I*_Na_). Spike amplitude depended on the availability of *I*_Na_ and spike kinetics on the membrane time constant, and the spikes could be largely suppressed by TTX. Membrane resistance fluctuated rapidly and strongly, repeatedly changing over the course of recordings and causing closely correlated fluctuations in resting membrane potential. In a minority of cells, we found delayed secondary *I*_Na_-like inward currents characterized by comparatively small amplitudes and slow kinetics, which produced secondary depolarizing spikes. Up to three consecutive delayed inward current waves were detected. These currents could be rapidly and reversibly augmented by applying L-type Ca^2+^ channel blocker nifedipine to diminish influx of calcium and thus increase gap junctional conductance.

**Conclusions:**

This work shows, for the first time, that *I*_Na_ and *I*_Na_-mediated voltage spikes can spread laterally through gap junctions in the monolayer of cells that are traditionally considered non-excitable. Our findings support a potential role of the RPE that goes beyond giving homeostatic support to the retina.

**Supplementary Information:**

The online version contains supplementary material available at 10.1186/s12915-023-01559-5.

## Background

Retinal pigment epithelium (RPE) is adjacent to photoreceptors in the eye and critical for normal retinal functioning. RPE secretes growth factors; phagocytoses the exhausted fragments of photoreceptor outer segments (POS); regenerates the visual pigment; maintains ionic homeostasis of the sub-retinal space by transporting ions, water, and metabolic end products between the sub-retinal space and the blood; serves as a barrier between the bloodstream and the retina; absorbs light; and protects the retina from photo-oxidation [1, 2]. A loss or failure of any RPE function can have pathologic consequences in the retinal tissue leading to photoreceptor degeneration and even blindness [2, 3]. Several of these functions depend on ion channels expressed in the plasma membrane [2, 4–10] .

The channelome of RPE is large and includes many ligand- and voltage-activated channels with functional verification to at least TRP (TRPC1, 4; TRPV2); inwardly (Kir4.1, 6.2, 7.1) and outwardly rectifying voltage-activated K^+^ channels (K_V_1.2, 1.3, 1.4, 4.2; KCNQ1, 4, 5); Ca^2+^-activated K^+^ channels (BK); voltage-activated Ca^2+^ channels (Ca_V_1.2, Ca_V_1.3, Ca_V_3.1, Ca_V_3.2); Cl^−^ channels (ClC-2, best-1, cystic fibrosis transmembrane conductance regulator (CFTR)); connexin channels; and voltage-gated Na^+^ channels [5–8, 11–18]. Yet, RPE cells are not neurons; they are traditionally considered to have passive intrinsic properties, similarly to other non-excitable cells. Is this large channelome with several voltage-activated channels present in the RPE only to support its homeostatic activity?

This study was motivated by the question raised above as well as the recent discovery of voltage-activated Na^+^ channels in the RPE and their prominent role in the phagocytosis of POS [4]. Immunolabeling and mass spectrometry experiments revealed the expression of nine alpha-subunits of voltage-activated Na^+^ channels (Na_V_1.1, Na_V_1.3 to Na_V_1.9, and Na_X_), especially Na_V_1.4, Na_V_1.6, and Na_V_1.8 [4]. It was shown that exposure of RPE to tetrodotoxin (TTX) both in culture and ex vivo in the mouse, and also silencing the expression of Na_V_1.4 with shRNA, can suppress phagocytosis, raising a possibility that electrogenic function of these channels might contribute to phagocytosis [4]. By demonstrating the presence of Na^+^ channels also in the mouse RPE, these results contradict conclusions of earlier studies, in which inward Na^+^ current (*I*_Na_) was observed but attributed to the neuronal trans-differentiation of cultured primary RPE [16, 19].

Fast depolarizing voltage responses (“spikes”) mediated by *I*_Na_ have been previously described in the conventional non-excitable cells, including astrocytes and rabbit pigmented ciliary body epithelial cells [20–23]. In the study of cultured neonatal rat RPE, Botchkin and Matthews reported action potential-like depolarizing spikes [16]. However, although many studies have analyzed ionic currents in various RPE cells, our understanding of membrane properties and voltage responses is still incomplete.

Development of functionally mature human embryonic stem cell-derived RPE (hESC-RPE) holds promise for prospective transplantation treatments of RPE-dependent retinal degenerative diseases [24–26]. The hESC-RPE cell lines express RPE-specific genes and proteins, exhibit a hexagonal cobblestone morphology with appropriate polarization and barrier properties, strong pigmentation, and perform phagocytosis similarly to human fetal RPE [5, 27–30]. This functionality and promising outlook of hESC-RPE transplantation necessitate comprehensive study of their electrophysiological properties. In this study, by using the patch-clamp technique, we investigated membrane response properties of hESC-RPE cells in the monolayer in physiological conditions at body temperature. We describe the properties of spikes, a spontaneously and rapidly fluctuating leak conductance, and a previously unreported phenomenon of multiple waves of inward currents caused by signal spreading via gap junctions.

## Results

### Ionic currents in hESC-RPE

**Fig. 1:**
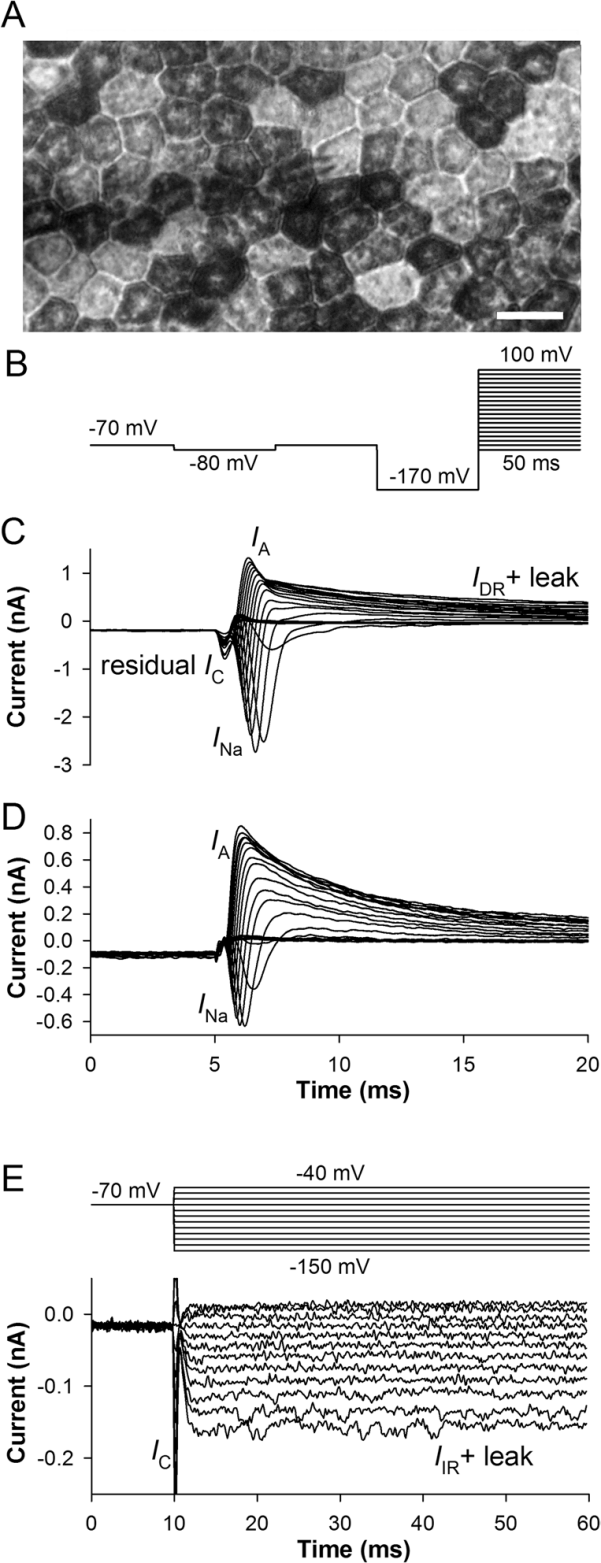
Ionic currents in hESC-RPE and their variability. **A** Bright-field microscopy image of hESC-RPE monolayer with the patch pipette attached to the cell. Scale bar is 10 μm. **B** Experimental protocol to record *I*_Na_; a 50-ms pulse to −80 mV was given from a holding potential of −70 mV to measure leak current and membrane capacitance; a 50-ms pre-pulse to −170 mV was used to recover *I*_Na_ before stimulating with 50-ms testing pulses in 10 mV increments from −80 to 100 mV. **C**,** D** Examples of whole-cell currents recorded from two cells; *I*_C_, residual capacitive current; *I*_Na_, voltage-activated Na^+^ current;* I*_A_ and *I*_DR_, the transient and sustained outward voltage-activated K^+^ currents, respectively. **E** An example of sustained hyperpolarization-activated inward current (*I*_IR_)

We studied electrophysiological properties of pigmented, cobblestone hESC-RPE cells (Fig. 1A) in whole-cell patch-clamp experiments by recording from 707 cells in voltage clamp mode; in 181 of these cells, additional current-clamp measurements were performed. There was a substantial variability in the ionic currents between cells (Fig. 1C,D). *I*_Na_ of variable amplitude (see *I*_Na_ in Fig. 1C and D for comparison) was detected in the majority (~98%) of cells using the recording protocol shown in Fig. 1B. The transient (*I*_A_) and sustained (delayed rectifier, *I*_DR_) voltage-activated K^+^ currents were expressed at dissimilar ratios (Fig. 1C, D). Similarly, leak conductance varied from cell to cell. Sustained hyperpolarization-activated inward current (*I*_IR_, Fig. 1E), possibly mediated by K^+^ inward rectifier channels, was found in the majority (~80%) of cells (Fig. 1D) [6].

**Fig. 2:**
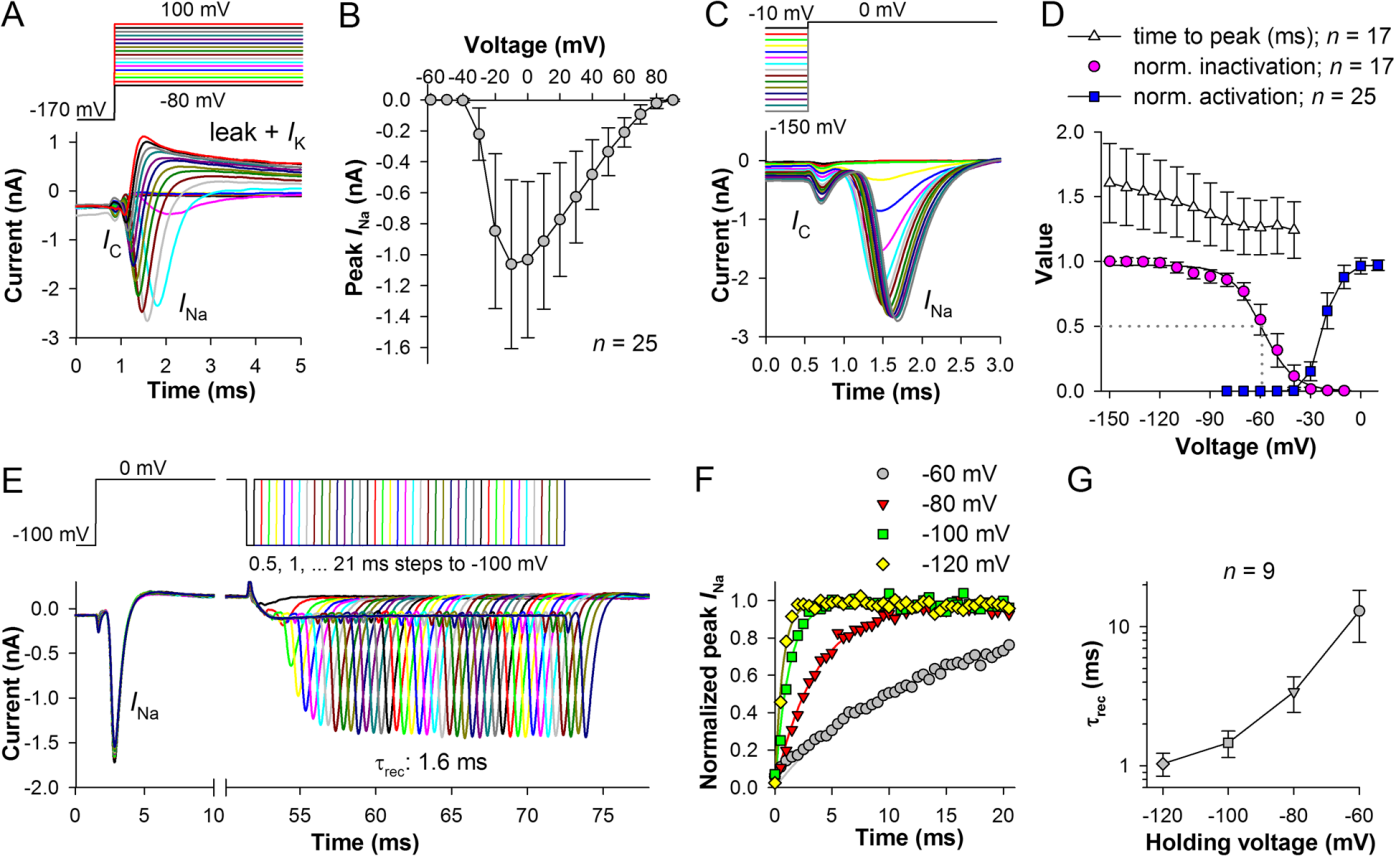
Kinetics of voltage-activated Na^+^ currents (*I*_Na_). **A** Typical *I*_Na_; *I*_C_, residual capacitive current; *I*_K_, the putative voltage-activated K^+^ current. **B** Average current-voltage (*I*–*V*) relationship for *I*_Na_ (*n* = 25); here and elsewhere error bars are s.d. (Additional file 2: Table S1). **C** Steady-state inactivation (SSI) was studied by evoking *I*_Na_ at 0 mV after 50 ms conditioning pre-pulses from −150 to −10 mV. **D** Voltage dependencies of the average peak *I*_Na_ (circles) and its time to peak (triangles) from SSI experiments (*n* = 17), and voltage dependence of the normalized peak *I*_Na_ conductance (*n* = 25, squares). SSI data were divided by the amplitude of *I*_Na_ after the pre-pulse to −150 mV; in the figure, average values were fitted with a sigmoidal equation. Conductance at each membrane potential *V* was calculated using peak *I*_Na_ from *I*–*V* relations as in **B** and equation *g*_Na_(*V*) = *I*_Na_/(*V* − *E*_rev_), where *E*_Na_ = 85.8 mV (Additional file 2: Table S1). **E** Recovery of *I*_Na_ from inactivation. *I*_Na_ was evoked and inactivated by 50-ms pre-pulses to 0 mV, after which hyperpolarizing pulses of increasing duration ranging from 0.5 to 21.0 ms were applied to recover *I*_Na_; this was followed by a testing pulse to 0 mV. In these experiments, the holding potential between the trials and the recovery potential were the same. **F** By plotting peak *I*_Na_ during recovery from inactivation against the hyperpolarizing pulse duration and then fitting the data with a first-order exponential rise-to-maximum equation, recovery time constants (*τ*_rec_) can be obtained; examples are from the same cell as in **E**. **G** Voltage dependence of average recovery time constants (Additional file 2: Table S1)

The ultra-fast transient depolarizing *I*_Na_ is conducted by several Na^+^ channel types [4]. Figure 2 describes the essential aspects of *I*_Na_ in hESC-RPE at the physiological temperature of 33–35°C. A typical *I*_Na_ is shown in Fig. 2A. Its amplitude was determined by subtracting the current after the end of the preceding capacitive current (*I*_C_) from the negative peak amplitude. At positive voltages, precise measurement of *I*_Na_ was often complicated due to progressive superposition of the partly compensated and slightly fluctuating *I*_C_ with *I*_Na_ as the activation kinetics of *I*_Na_ accelerated with depolarization. However, this complication was usually not present at voltages below 0 mV. *I*_Na_ activation threshold was in between −40 and −30 mV. The average current-voltage (*I*–*V*) relation for a group of cells without capacitive artifacts is shown in Fig. 2B. It can be seen that the current disappears by +80 mV, consistently with the +86 mV reversal potential for Na^+^ created by the recording solutions (see “Methods”).

In addition to *I*_Na_, RPE can express voltage-activated Ca^2+^ channels that mediate an inward Ca^2+^ current with voltage dependencies similar to those of *I*_Na_ but slower kinetics [31, 32]. Because calcium currents in hESC-RPE appear to be very small and mostly non-inactivating, requiring replacement of Ca^2+^ ions by Ba^2+^ ions in the external solution for measurement [5], we did not investigate them specifically. However, we verified the consistency of *I*_Na_ characteristics without the contribution of K^+^ and Ca^2+^ channels using Cs^+^-based intracellular solution and 10 μM nifedipine extracellularly (Additional file 1. Fig. S1) [33].

Next, we determined voltage dependencies of several *I*_Na_ parameters. Availability of *I*_Na_ was investigated using a steady-state inactivation (SSI) protocol consisting of 50 ms conditioning pulses between −150 and −10 mV followed by a test pulse to 0 mV (Fig. 2C). As can be seen from the gradually decreasing *I*_Na_ during the test pulse, exposure to voltages in the range from *ca.* −120 mV to the *I*_Na_ activation threshold caused closed-state inactivation (Fig. 2C, D). The half-inactivation potential was −57.8 ± 4.4 mV (Fig. 2D). In particular, 27% of *I*_Na_ was on average available after a brief exposure to −50 mV and 11% after holding at −40 mV. Interestingly, *I*_Na_ time to peak decreased steadily with increasing voltage of the conditioning pulse, from 1.60 ± 0.30 ms after holding at −150 mV to 1.27 ± 0.21 ms after holding at −70 mV (*P* = 10^−11^, paired *t*-test, Fig. 2C, D), indicating voltage-dependent facilitation of *I*_Na_. This is probably caused by accumulation of Na^+^ channels in the conformation states that promote both rapid opening and steady-state inactivation. The overlap between the voltage dependencies of activation (obtained from *I*–*V* relations, the half-activation potential of 23.1 ± 1.6 mV) and inactivation indicated that a small window current might exist around −35 mV (Fig. 2D).

Recovery from inactivation was evaluated at four membrane potentials (Fig. 2E–G). After a 50-ms pulse to 0 mV to activate and inactivate *I*_Na_, the cell was exposed for variable durations to the test potential, from 0.5 to 21.0 ms in 0.5-ms increments. The resulting response series allowed measuring the time constant of recovery by fitting the time course of peak *I*_Na_ increase with a first-order exponential rise-to-maximum equation (Fig. 2E, F). Time courses of recovery with exponential fits are shown for one cell in Fig. 2F. Figure 2G demonstrates the average voltage dependence of recovery time constants. *I*_Na_ recovered with the time constant of 1.05 ± 0.26 ms at −120 mV but only 16.9 ± 6.7 ms at −60 mV.

### Spikes in the current-clamp experiments

The presence of *I*_Na_ in hESC-RPE raises an immediate question about the capability of RPE cells to generate transient depolarizations, such as action potentials or spikes. To further investigate their electrophysiological properties in general and voltage spiking in particular, we used several current-clamp protocols, all of which contained a hyperpolarizing current injection to recover *I*_Na_ prior to a test stimulus. The duration of such hyperpolarizing pulses was set to 50 ms and amplitude varied depending on the membrane resistance in order to hyperpolarize membrane potential to below −100 mV to quickly recover most of *I*_Na_ (Fig. 2G).

**Fig. 3:**
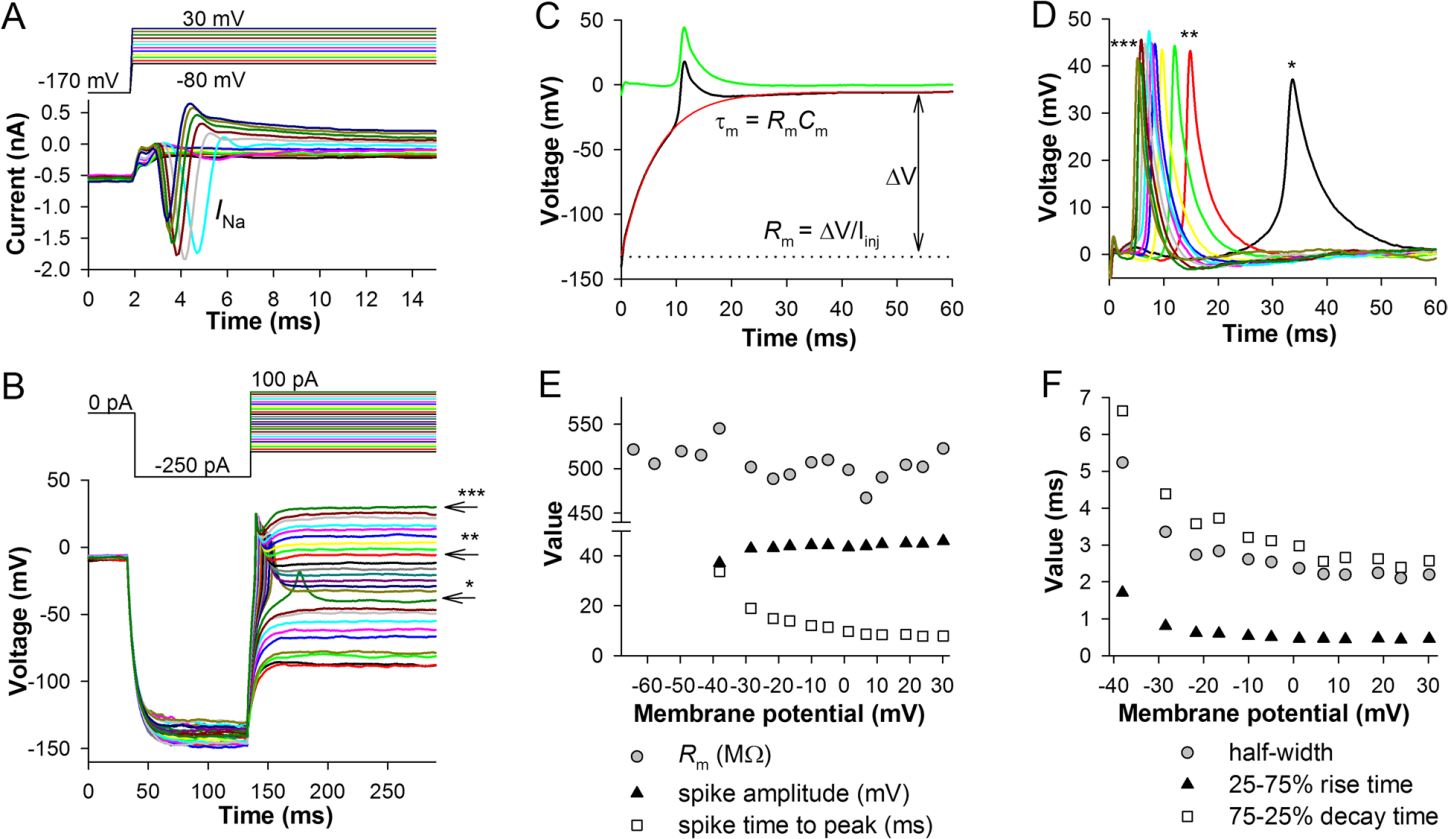
Fast sodium spikes in current-clamp experiments. **A** Example of voltage clamp recordings. **B** Spikes in a current-clamp experiment were evoked by first hyperpolarizing the cell with 100-ms current injections to below −100 mV and then applying depolarizing pulses as indicated. **C** Separation of a spike (green trace) from the voltage response (black); the region containing a spike was excised and the remaining trace fitted with a first-order exponential rise-to-maximum equation (red); subtracting the traces elicited the spike. Membrane resistance (*R*_m_) was determined from the amplitude of the voltage response (Δ*V*) to the injected current (*I*_inj_); membrane time constant (*τ*_m_) obtained by the fitting was used to calculate membrane capacitance (*C*_m_) as indicated. **D** Examples of spikes; asterisks correspond to the marked traces in **B**. **E** Voltage dependencies of membrane resistance, spike amplitude, and spike time to peak in the same experiment; note that the membrane potential refers to the steady-state voltage reached *after* membrane charging is finished, which is in most cases higher than the potential at which the spike is initiated. **F** Voltage dependencies of spike parameters: half-widths, 25–75% rise and 75–25% decay times

Figure 3A shows ionic currents and Fig. 3B voltage responses to a series of depolarizing current pulses recorded in the same cell. Spikes appeared when membrane was depolarized to above −40 mV, with their times to peak decreasing with depolarization. Another important feature was that in the absence of spikes, the RPE membrane behaved like a passive electrical circuit, i.e., the time course of charging could be easily fitted with a first-order exponential function (see the stable membrane resistance in Fig. 3E).

To study the properties of spikes, it was necessary to separate them from the underlying voltage responses. We used a procedure described in Fig. 3C. The spike distorts the rising phase of the voltage response causing a deviation from the uniform exponential rise to maximum. By removing the spike-containing part of the trace and fitting the rest with a first-order exponential rise-to-maximum equation, an estimate of a response without the spike can be obtained (Fig. 3C, red trace). By subtracting it from the original trace, the spike waveform is acquired (Fig. 3C, green trace). Spikes from Fig. 3B are shown in Fig. 3D and their properties are described in Fig. 3E, F. Spike amplitude changed little with depolarization, unlike the amplitude of *I*_Na_ (Fig. 3E). In contrast, spike time to peak decreased strongly with depolarization, similarly to changes in the time to peak of *I*_Na_ (Fig. 3A), albeit in a much wider temporal range due to the relatively slow membrane charging in the current-clamp experiments. Kinetics of spikes, including the 25–75% rise and decay times, also accelerated, resulting in a gradual narrowing of spikes with depolarization (Fig. 3F). Because membrane was passive in the studied voltage range, these differences could be attributed to voltage dependence of *I*_Na_ kinetics.

**Fig. 4:**
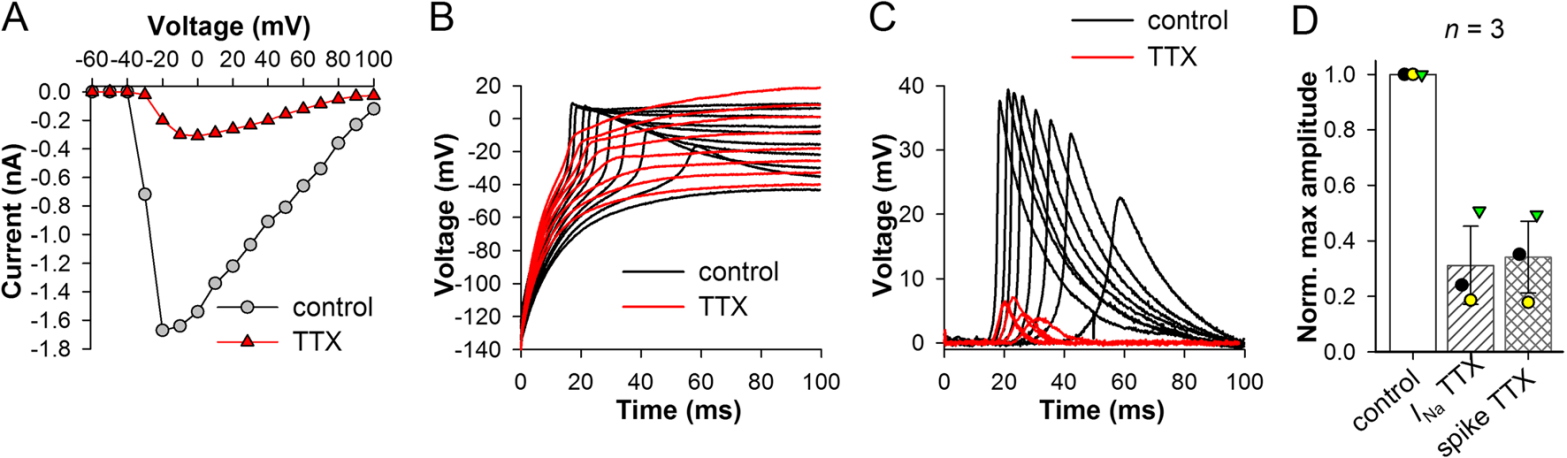
Effect of TTX on *I*_Na_ and spikes. **A** *I*–*V* relations from an experiment involving inhibition of *I*_Na_ with 10 μM TTX. **B** Traces of current-clamp recordings in control conditions with spikes visible and after application of TTX from the same cell as in **A**; data were obtained as described in Fig. 3B; stimuli not shown. **C** Isolated spikes from **B**. **D** Summary of TTX experiments. Amplitudes of the largest *I*_Na_ and spikes were measured in three cells before and after application of TTX; values in the presence of TTX were divided by control ones and averaged (Additional file 2: Table S1)

Although evidence suggested that spikes are caused by *I*_Na_, it was necessary to perform a definitive test. We used 10 μM TTX to block *I*_Na_ and evaluate changes in spikes (Fig. 4). TTX strongly suppressed *I*_Na_ (Fig. 4A). Simultaneously, spike amplitudes decreased dramatically and clearly in proportion to changes in the peak *I*_Na_ (Fig. 4B, C). This was observed in all three experiments (*P* = 0.02 for both *I*_Na_ and spike amplitude, paired *t*-test, Fig. 4D). These results unequivocally prove that spikes are mediated by *I*_Na_.

**Fig. 5:**
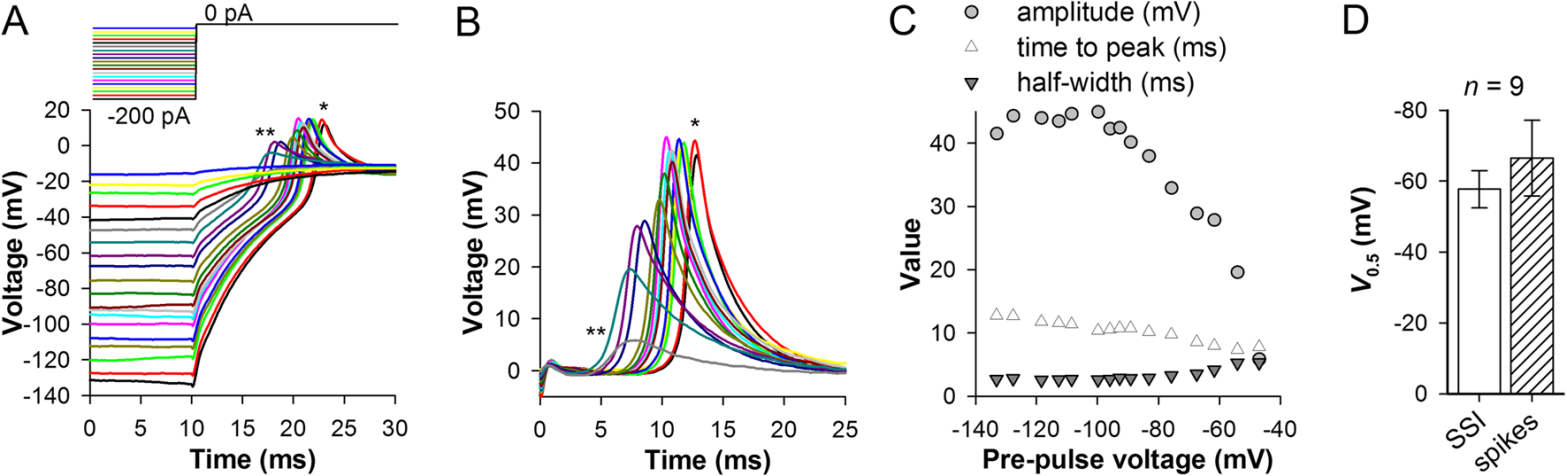
Voltage dependence of spikes. **A** Voltage responses of an RPE cell at resting potential after a series of 100-ms conditioning hyperpolarizing pre-pulses. **B** Isolated spikes from **A**, with asterisks indicating the correspondence between spikes in **A** and **B**. **C** Dependence of spike amplitude, time to peak and half-width on the pre-pulse voltage; data from **A** and **B**. **D** Comparison of the average half-inactivation potentials from SSI recordings and the average half-maximal amplitude potentials for spikes (both denoted with *V*_0.5_) in the same cells; the voltage dependencies were fitted with a sigmoidal equation (Additional file 2: Table S1).

Next, we investigated the voltage dependence of spikes. Figure 5A–C shows a representative experiment. The cell was stimulated with 100 ms hyperpolarizing conditioning current pulses of different amplitudes to obtain membrane potentials in the range from below −120 mV (to fully recover *I*_Na_) to about resting potential. The hyperpolarizing pulses were followed by relaxation to resting potential, which triggered the spikes, depending on the preceding membrane potential. The spike amplitude and time to peak decreased with depolarization, whereas the half-width increased (Fig. 5C). The decrease in amplitude was due to closed-state inactivation of *I*_Na_ as the consistent average half-inactivation potential for *I*_Na_ and the half-maximal spike amplitude potential indicate (Fig. 5D). The decrease in the time to peak was due to a comparatively fast membrane charging toward the *I*_Na_ activation threshold from more depolarized conditioning potentials.

Because voltage-gated K^+^ conductances can condition spikes caused by *I*_Na_, and also because activation of delayed rectifier K^+^ conductances by a depolarizing current injection can cause spike-like voltage transients [34], we correlated spike sizes and half-widths with the amplitudes of *I*_DR_ and *I*_A_. The currents were measured after a 50-ms pre-pulse to −170 mV at the nominal voltage pulse to +100 mV (see Fig. 1B).* I*_A_ was measured as the difference between the peak outward current and the sustained current at the end of 50-ms pulses. *I*_DR_ was determined at the end of 50 ms pulses, with leak subtracted. We found a weak positive correlation (*r* = 0.2, *P* = 0.019, *n* = 140) between *I*_A_ and spike amplitudes but no correlation between *I*_DR_ and spike amplitudes. No correlations could be found between *I*_A_ and *I*_DR_ amplitudes, on the one hand, and spike half-width, on the other hand.

**Fig. 6:**
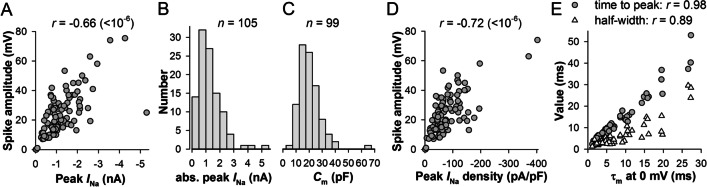
Spikes, *I*_Na_ and membrane time constant. **A** Correlation between the maximal spike and *I*_Na_ amplitudes; *I*_Na_ was measured from a recording immediately preceding the current-clamp. **B**, **C** Distribution of maximal *I*_Na_ amplitudes (**B**) and *C*_m_ values (**C**); the distributions are not Gaussian (*P* < 0.001, Shapiro-Wilk normality test). **D** Correlation between the maximal spike amplitudes and *I*_Na_ densities; *I*_Na_ density was obtained by dividing peak *I*_Na_ with the corresponding *C*_m_ value. **E** Plots of spike time to peaks and half-widths against membrane time constants; the spikes were obtained from voltage responses reaching the level of ca. 0 mV (−0.9 ± 2.7 mV, *n* = 60) from voltages below −100 mV (−131 ± 21 mV, *n* = 60); the correlations were characterized by *P* values of < 10^−6^

In contrast, the maximal spike amplitude correlated strongly with the peak *I*_Na_, with *r* = −0.66 (Fig. 6A, *n* = 95). Figure 6B clearly shows that the distribution of peak *I*_Na_ was not Gaussian, with a median of −1140(−765:1635) pA (*n* = 105).

However, membrane charging by *I*_Na_ during the onset of the spike depends not only on *I*_Na_ amplitude but also on its density. Figure 6C shows that capacitance, a proxy for cell membrane area, varied substantially in our experiments. The distribution was also not Gaussian, with a median of 17.1(11.6:21.9) pF (*n* = 99). The correlation between the maximal spike amplitude and *I*_Na_ density obtained by dividing peak *I*_Na_ by *C*_m_ (Fig. 6D) was −0.72, stronger than that in Fig. 6A.

Speed of membrane charging varied between cells and often in the same cell during prolonged recordings (see next section). To test how variability in membrane time constant could affect the spike, we correlated spike properties to the corresponding time constants. Although membrane time constant (*τ*_m_) did not depend on membrane potential, *I*_Na_ did, and so in the following analysis we used spikes obtained from voltage response traces with sustained potentials at around 0 mV and evoked from voltages below −100 mV to minimize closed-state inactivation of *I*_Na_. There was no correlation between spike amplitude and *τ*_m_ (*r* = 10^−2^), i.e., the speed of membrane charging had no effect on the spike size. However, a nearly unity (0.98) correlation was discovered between *τ*_m_ and spike’s time to peak (Fig. 6E), and a less strong correlation (*r* = 0.89) was observed between *τ*_m_ and spike’s half-width (Fig. 6E). The latter correlation was caused by strong dependencies on *τ*_m_ of spike’s 25–75% rise and decay times, with the corresponding correlation coefficients of 0.74 and 0.88 (for both, *P* < 10^−6^; *n* = 60).

**Fig. 7:**
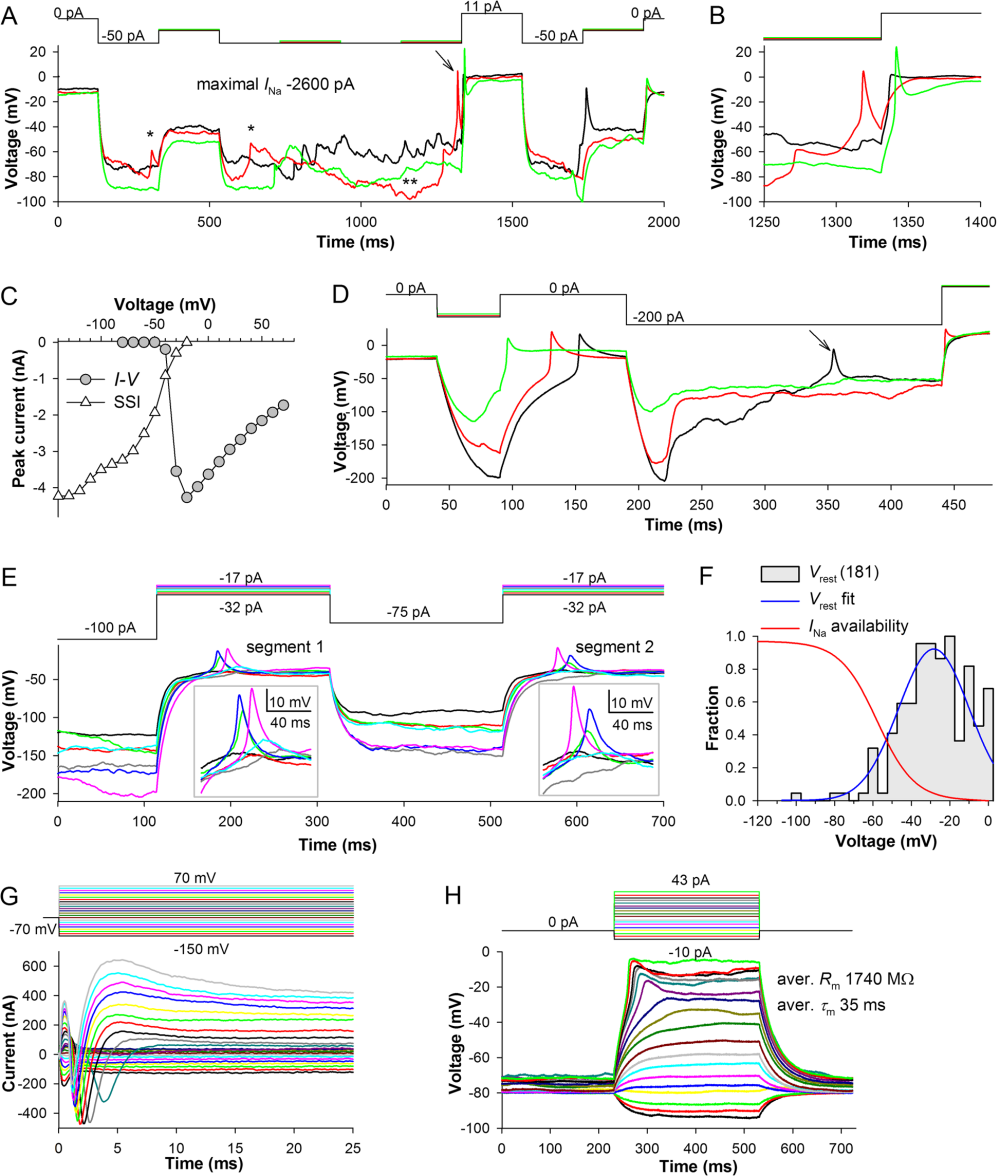
Stochasticity of spike onset. **A** Spikes can occur apparently spontaneously when the cell is hyperpolarized by current injection; asterisks denote two particularly sharp depolarizing transients and the double asterisk the region of spontaneous hyperpolarization preceding the actual spike (arrow). **B** Magnified spike-containing segment from (**A**). **C** *I*–*V* and SSI relations for another cell characterized by a large *I*_Na_ with a comparatively negative *I*_Na_ activation threshold. **D** Parts of three voltage traces from a current-clamp recording, with a spike at 360 ms on the black trace. **E** Variability in spike properties in the *I*_Na_ activation threshold region. **F** Distribution of minimal resting potentials, its Gaussian fit, and the *I*_Na_ availability curve obtained by fitting the SSI data from Fig. 2D; the *V*_rest_ histogram data were divided by the maximum value. **G**, **H** Ionic currents (**G**) evoked from a holding potential of −70 mV in an RPE cell with a very negative *V*_rest_ and the associated current-clamp recording (**H**)

Although we did not register spontaneous spikes when membrane potential was not hyperpolarized by current injections, there is evidence that they can be evoked if membrane potential transiently decreases to recover a sufficient fraction of *I*_Na_. Figure 7A demonstrates three traces recorded in a current-clamp experiment. The red trace contains a spike (arrow) triggered by a sharp spontaneous depolarization after a brief hyperpolarization (two asterisks). The same trace also contains episodes of rapid depolarization (asterisks), which, however, failed to trigger spikes. Immediately after the spontaneous spike, the hyperpolarizing current pulse was switched off and the cell depolarized to about 0 mV (Fig. 7B). Because available Na^+^ channels were inactivated during the spike, no new spike could be elicited, in contrast to the other two traces (black and green), where spikes did appear.

In another experiment, *I*_Na_ was large, had a low activation threshold, and its substantial fraction (~20%) was available after a 50 ms exposure to −40 mV (Fig. 7C, white triangles). Figure 7D shows three voltage traces from the associated current-clamp recording. A large spontaneous spike can be seen on the black trace (arrow). Its activation threshold was about −60 mV and, by comparison with two other traces characterized by the similar membrane potential, it appears that the spike was evoked because a substantial fraction of *I*_Na_ recovered during the preceding hyperpolarization between 220 and 310 ms.

Figure 7E illustrates the stochasticity of spike amplitudes and kinetics when they were triggered around the *I*_Na_ threshold potential in another RPE cell. Seven traces are shown. Clear spikes can be seen in four traces during two depolarizing segments. No spike appeared in the black trace, although during the second depolarizing segment a slow transient was evoked. This trace was characterized by the most positive membrane potentials before the depolarizing pulses, so that 97% of the maximal *I*_Na_ was available before the first and 84% before the second depolarizing segment (values from the associated SSI recording). Similarly, no spike was evoked during the red trace characterized by even more hyperpolarized potentials. Green and cyan traces almost coincided with each other and with the red trace in their pre-depolarization membrane potential but only the green trace had clear spikes. Likewise, gray and blue traces were quite similar in their membrane potentials, but spikes appeared only on the blue trace. Two spikes can be seen in the pink trace but their timing relative to other spikes was different during the two depolarizing segments.

Although we found no clear spikes at resting membrane potential (*V*_rest_) in our recordings, any residual *I*_Na_ persisting at *V*_rest_ or recovered by a preceding hyperpolarizing fluctuation of membrane potential can plausibly amplify the next rapid depolarizing fluctuation. The resulting transient might resemble the smallest dark gray spikelet in Fig. 5B or the fast depolarizing spikelets on the red and black traces in Fig. 7A. To estimate the available fraction of *I*_Na_ at rest in the experimental group, we plotted the histogram (Fig. 7F) of the most negative *V*_rest_ values obtained in each cell in the absence of current injection (e.g., from the green trace in Fig. 7A at 0 ms or the black trace in Fig. 9 at 0 ms). The average *V*_rest_ was −29 ± 18 mV (181 cells), which would become −42 mV after the liquid junction potential error correction. The histogram was fitted with a Gaussian equation (blue trace) and superimposed on the SSI fitting curve from Fig. 2D (red trace). In the minority of cells characterized by *V*_rest_ below −50 mV, more than 40% of *I*_Na_ could be available. However, in the majority of cells, *I*_Na_ at *V*_rest_ was inactivated almost completely.

An example of a cell with a very low *V*_rest_ is shown in Fig. 7G, H. The currents were recorded from a holding potential of −70 mV. Figure 7G shows comparatively large *I*_Na_, *I*_A_, *I*_DR_, *I*_IR_, and a small leak current in the vicinity of *V*_rest_. In the current-clamp measurements, *V*_rest_ occasionally reached −80 mV, indicating the availability of a large fraction of *I*_Na_. Consequently, upon stimulation with depolarizing current injections, spikes were elicited after crossing the *I*_Na_ activation threshold at about −40 mV. The low *V*_rest_ was associated with very high average *R*_m_ of 1730 MΩ and membrane time constant of 35 ms, which could explain the stability of membrane potential and absence of fluctuations.

### Dynamic changes in membrane resistance

**Fig. 8:**
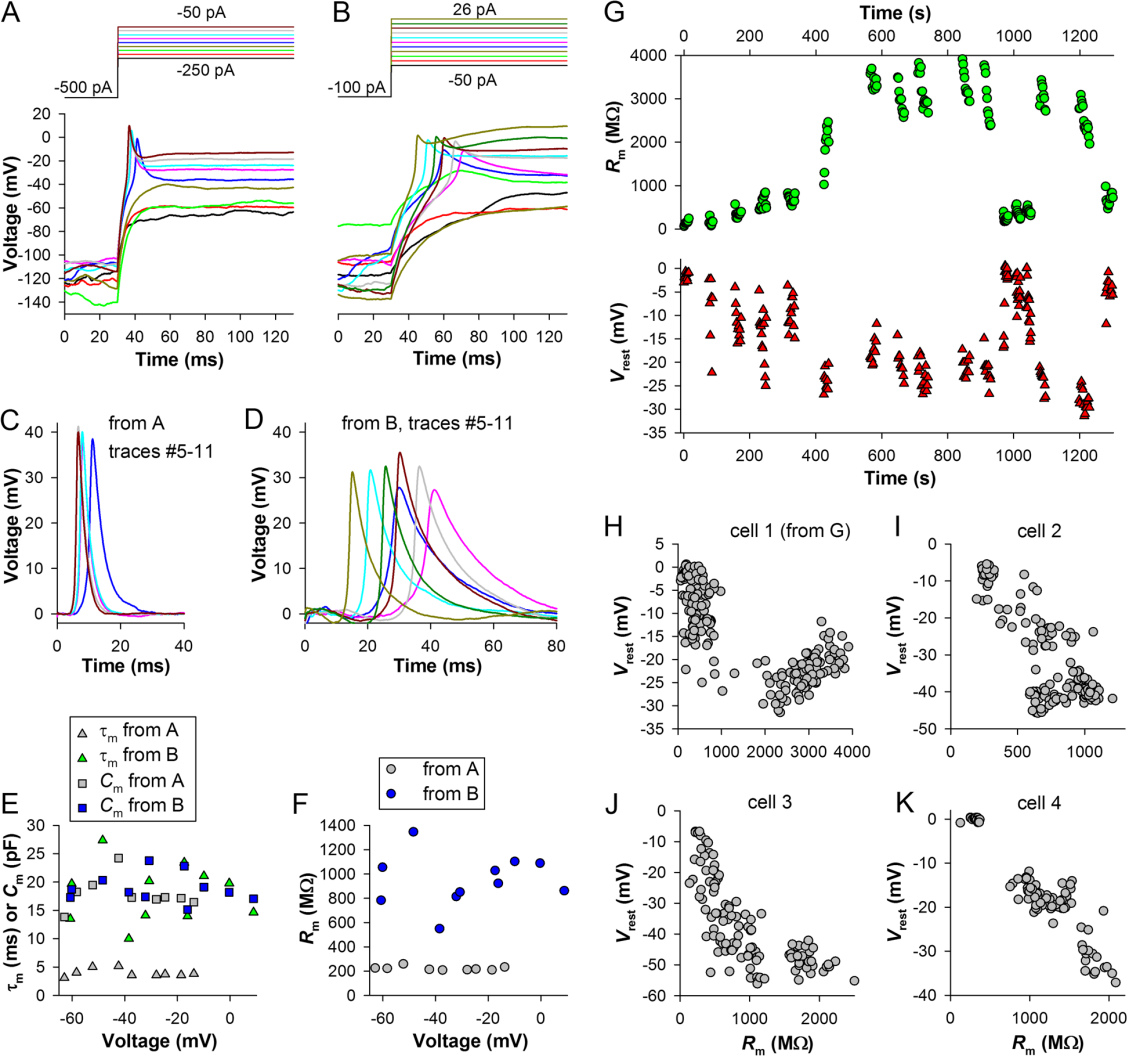
Spontaneous changes in leak conductance and its effects. **A**, **B** A recording with a low *R*_m_ (**A**) was followed by a recording with a relatively high *R*_m_ (**B**); **C**, **D** Spikes from the low- (**C**) and high-resistance (**D**) recordings; color coding is the same as in **A** and **B**, respectively. **E** Dependencies of membrane time constants and capacitances on the response voltage immediately after the spike. **F** Dependencies of membrane resistances on the response voltage immediately after the spike; **A–F**, data from the same cell. **G** Time courses of changes in *R*_m_ (top) and resting potential *V*_rest_ (bottom) during one experiment. **H**–**K** Correlations between *R*_m_ and *V*_rest_ during similarly prolonged recordings in 4 cells

Figure 3 shows current-clamp recordings characterized by relatively stable *R*_m_ (Fig. 3E) and *V*_rest_ values (Fig. 3B). However, such stable recordings were exceptions rather than a rule because *R*_m_ and *V*_rest_ tended to change continuously. Figure 8A, B demonstrates parts of two recordings from the same cell, both displaying large spikes (Fig. 8C, D). During the recording in Fig. 8A, the cell had relatively small *τ*_m_ (Fig. 8E) and *R*_m_ (Fig. 8F), on average 222 MΩ. To charge its membrane potential to below −100 mV, a comparatively large hyperpolarizing current of −500 pA was needed (top).

In contrast, the recording in Fig. 8B was characterized by large *τ*_m_ (Fig. 8E) and *R*_m_ (Fig. 8F), and a small −100 pA current hyperpolarized membrane potential to below −100 mV. The relatively low-resistance green trace in Fig. 8B illustrates sudden changes in *R*_m_ during the recordings (each trace is a part of a 1-s recording). Importantly, while the average *R*_m_ and membrane time constant increased by threefold, the average *C*_m_ did not change (Fig. 8E), supporting the validity of our estimation procedure (Fig. 3C). The differences in the properties of spikes (Fig. 8C, D) were consistent with the findings shown in Fig. 6E: changes in membrane time constant had a small effect on the spike amplitude but strongly altered their times to peak, half-widths, and kinetics.

Top plot of Fig. 8G shows an example of periodic changes in *R*_m_ over the course of a prolonged experiment consisting of many current-clamp recordings. *R*_m_ varied from 60 to 3900 MΩ, which corresponded to changes in membrane conductance from 17 to 0.26 nS. Comparison of time courses of changes in *R*_m_ and *V*_rest_ (Fig. 8G, bottom) indicated that *V*_rest_ depolarized when *R*_m_ decreased and vice versa. This implies that the fluctuating membrane leak was depolarizing, i.e., mediated by an inward cationic or outward anionic current. Figure 8H shows *V*_rest_ values plotted against the respective *R*_m_ values for the whole experiment in Fig. 8G, and Fig. 8I–K demonstrates similar plots for three more experiments. All results were consistent: *V*_rest_ was a function of membrane leak.

**Fig. 9:**
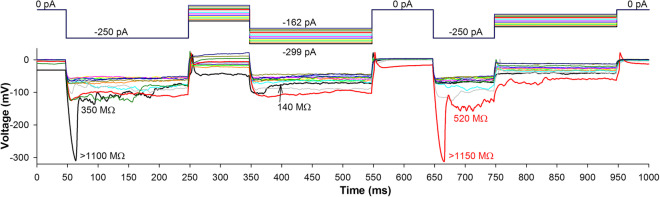
Sudden changes in leak conductance. While leak conductance can change gradually, in some cases its decrease occurs suddenly in the beginning of a hyperpolarizing pulse, manifesting in a sharp hyperpolarizing transient. *R*_m_ values in black font refer to the black trace and in red to the red trace

Although changes in *R*_m_ and *V*_rest_ usually occurred gradually, they were often facilitated by strong and sudden membrane depolarization or hyperpolarization. Figure 9 shows two examples of rapid hyperpolarization-induced decreases in *R*_m_. Over the course of the black trace, the cell was first characterized by a comparatively negative *V*_rest_ and high *R*_m_, >1100 MΩ. Even before the membrane potential had fully settled during a −250 pA current pulse, a sudden decrease in *R*_m_, to ~350 MΩ, happened at 62 ms. Eventually, after the decrease in *R*_m_ at ~370 ms, the cell acquired a resistance of 140 MΩ. Accordingly, *V*_rest_ increased from −32 mV at the trace onset to +2 mV at 650 ms. The second negative transient, with the corresponding *R*_m_ estimates, can be seen on the red trace at 670 ms.

### Multiple waves of excitation

**Fig. 10:**
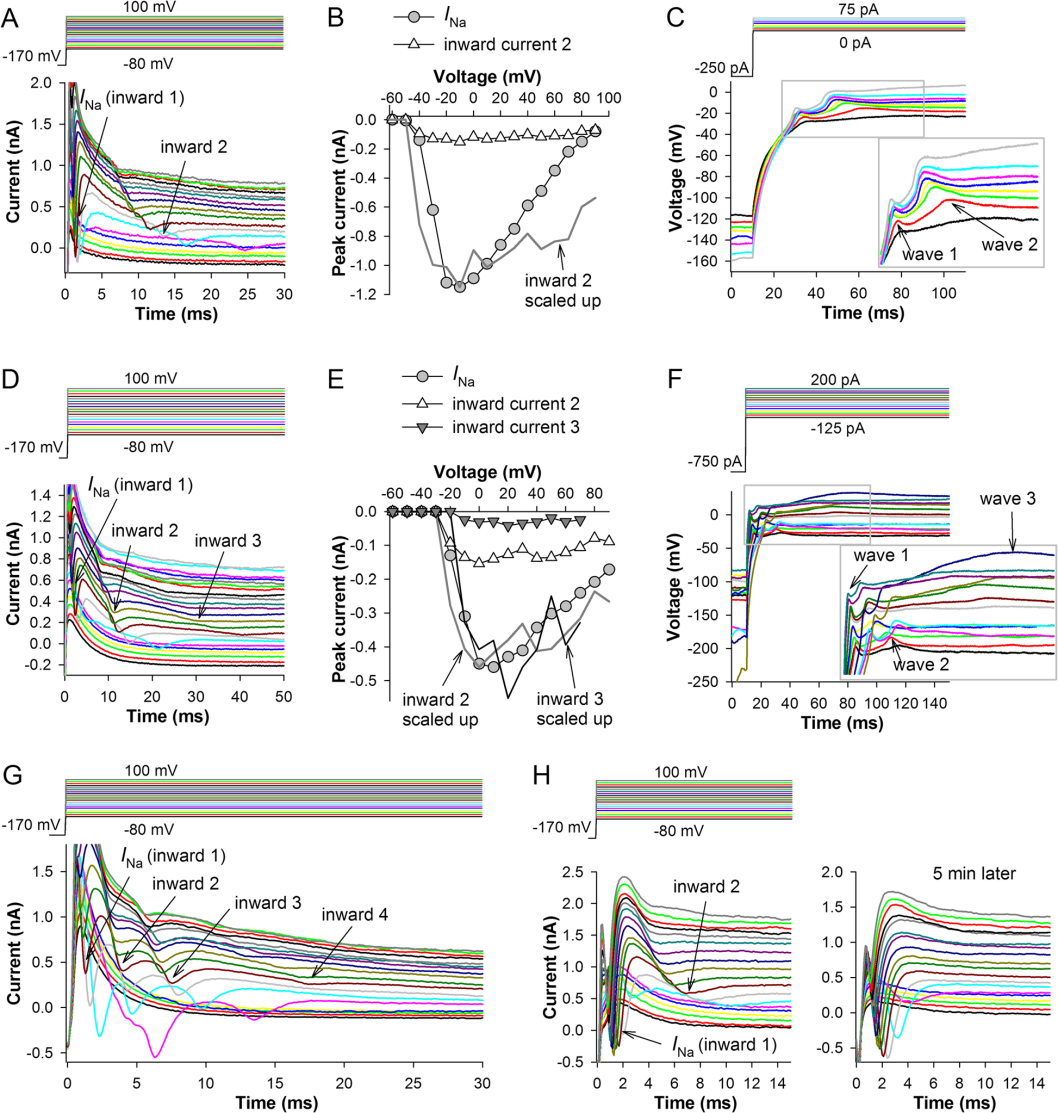
Multiple inward currents. **A** Voltage clamp recording with two depolarizing currents, a conventional *I*_Na_ (inward 1) and a relatively small and slow delayed inward current (inward 2). **B** The *I*–*V* relations for the two currents and the scaled up inward 2 current; inward 2 current was isolated by (1) removing the delayed current-containing part of the trace, (2) fitting the remaining trace between 5 and 30 ms with a polynomial equation, and (3) subtracting the fitting curve from the original trace. **C** A current-clamp recording from the same cell with two types of voltage spikes that correspond to the currents in **A**. **D**–**F** A recording from another cell, **D** with three waves of inward currents, **E** the corresponding *I*–*V* relations, and **F** the current-clamp recording. **G** Example of four inward currents from a different cell. **H** An example of recordings where the delayed inward current (inward 2, left) disappeared spontaneously after 5 min of recordings (right)

In 19 out of 707 studied cells, in addition to the conventional *I*_Na_, we found anomalous secondary depolarizing currents (Fig. 10A) characterized by relatively large times to peak, with slow onsets and inactivation kinetics. The currents were clearly voltage-dependent.

To investigate the properties of these delayed inward currents, we separated them from the outward current using a procedure similar to that we used to isolate spikes (Fig. 3C). Comparison of the *I*–*V* relations for *I*_Na_ and the delayed inward current showed that the extrapolated reversal potential of the new current should be extremely positive (*ca.* +200 mV, Fig. 10B). In the current-clamp experiments, the delayed inward current caused secondary spikes (Fig. 10C). Similarly to the closed-state inactivation of *I*_Na_ (Fig. 2D), the delayed inward current is inactivated by pre-exposing the cell to the same range of membrane potentials.

**Fig. 11:**
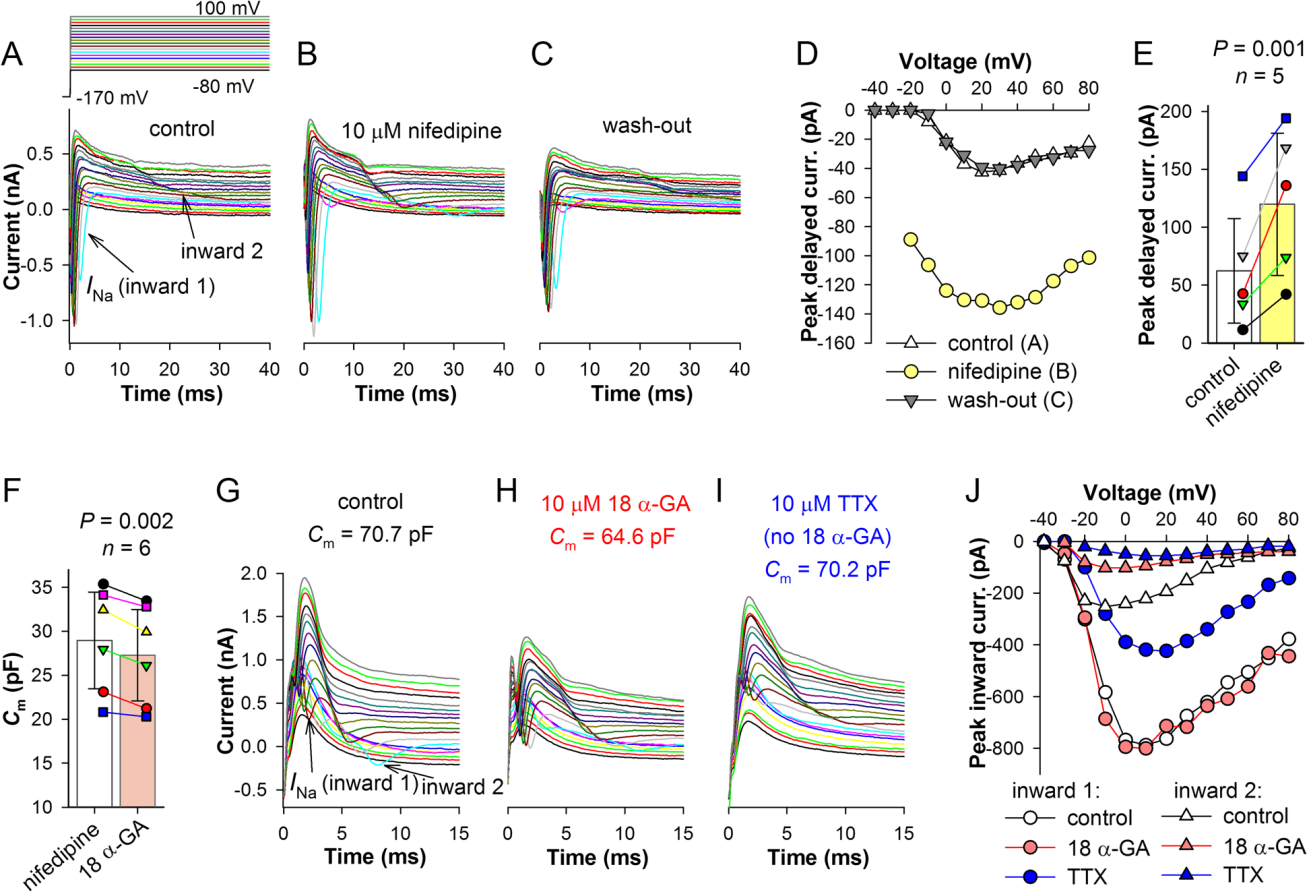
Effects of nifedipine and 18α-GA on the delayed inward current. **A**–**C** Voltage clamp recordings in control conditions (**A**), after application of 10 μM nifedipine (**B**), and after wash-out (**C**). **D** *I*–*V* relations for the delayed inward currents from **A** to **C** (inward 2). **E** Bar plot summarizes changes in the peak delayed inward current in 5 cells in the presence of 10 μM nifedipine; paired *t*-test was used for the statistical comparison. **F** Cells were first exposed to 10 μM nifedipine and then, after wash-out of nifedipine, to 10 μM 18α-GA; bar plot shows changes in *C*_m_ (*n* = 6; *P* = 0.002, paired *t*-test). **G**–**J** Voltage clamp recordings in control conditions (**G**), after application of 10 μM 18α-GA (**H**), and, after wash-out, 10 μM TTX (**I**); **J** *I*–*V* relations for this experiment. In panels **F–J**, recordings were performed with Cs^+^ in the pipette solution

Surprisingly, detailed examination of several recordings characterized by the presence of the second inward current revealed additional delayed inward currents (Fig. 10D–G). In Fig. 10D, the third inward current (inward 3) was small and even more delayed and slower than the second one (inward 2), eliciting small and slow depolarizations (Fig. 10F). In this cell, the second inward current *I*–*V* relation (Fig. 10E) had apparently a similarly positive reversal potential as the delayed inward current in Fig. 10A, B, whereas the *I*–*V* relation for the third current could not be reliably estimated beyond +70 mV. It should be noted that the cells with delayed inward currents tended to have unusually high *C*_m_ values (see the prolonged membrane charging in Figs. 10A, D, G and 11A–C, G–I), which could not be compensated properly, hence the nominal voltage in *I*–*V* relations was probably a large overestimation. Membrane capacitance estimated in the voltage clamp recordings from the charge during the capacitive transient was on average 54(29:69) pF (*n* = 19) in the cells with the secondary delayed inward current but only 26(21:34) pF (*n* = 685) in the typical cells (*P* < 10^−3^, Mann–Whitney *U*-test).

Figure 10G shows voltage clamp recording from a different cell with four depolarizing inward currents. Three inward currents were present at all potentials, except the threshold one, where the conventional *I*_Na_ and the first delayed inward current partly merged. The inward current 4 in Fig. 10G was small and slow, resembling the Fig. 10D current, and appeared at more positive potentials than the inward currents 1–3.

The presence of multiple depolarizing currents rules out a possibility of their origin in the same cell. This phenomenon can only be explained by the spread of excitation in both voltage- and current-clamp experiments to the neighboring cells via open gap junction channels. Consistently with this hypothesis, we found that the delayed inward current can be regulated dynamically, as shown in Fig. 10H. In six cells, it disappeared completely within minutes after the start of voltage clamp recordings.

Furthermore, gap junction conductance can be increased by lowering internal calcium concentration and vice versa [35]. Because L-type Ca^2+^ channels are expressed in hESC-RPE and are partly open at their typically depolarized resting potentials [5], we attempted to alter the internal calcium homeostasis by blocking these channels with nifedipine, which at 10 μM abolishes most of L-type Ca^2+^ channel current [5]. We recorded from five cells exhibiting delayed inward currents. Surprisingly, delayed inward currents increased noticeably immediately after application of 10 μM nifedipine. Figure 11A–C shows the effect of nifedipine in one cell. The effect was fully reversible (Fig. 11C, D). Bar plot in Fig. 11E summarizes the results of the experiments: on average, the maximal delayed inward current increased by more than 2-fold.

If nifedipine facilitates opening of gap junctions, then this can manifest in changes of *C*_m_. However, because the gap junction conductance status in control cells is not known, we compared *C*_m_ in the presence of 10 μM nifedipine to that after wash-out of nifedipine and application of connexin 43 (Cx43) channel inhibitor 18α-glycyrrhetinic acid (18α-GA). Cx43 is the most abundant gap junction protein in human RPE [11, 17]. We used 10 μM concentration of 18α-GA, which, while only partly blocking gap junctions, does not cause irreversible effects [36]. Membrane capacitance measured in the voltage clamp decreased slightly but statistically significantly, from 29.0 ± 5.5 pF in the presence of nifedipine to 27.3 ± 5.2 pF after application of 18α-GA (*n* = 6; *P* = 0.002, paired *t*-test, Fig. 11F). It should be noted that these measurements were conducted on cells that displayed no delayed inward currents.

In one cell with a delayed inward current, application of 10 μM 18α-GA caused a decrease in *C*_m_ and a simultaneous decrease in the delayed current but did not affect the amplitude of the primary *I*_Na_ (Fig. 11G, H). When 18α-GA was washed out and replaced with TTX, *C*_m_ returned to the control value while the amplitudes of both inward currents decreased (Fig. 11 I). The results of this experiment summarized in Fig. 11J are consistent with the hypothesis that the delayed inward currents originate in the neighboring cells and are mediated by *I*_Na_, with signals spreading via gap junction channels.

## Discussion

In this work, we (1) described Na^+^-based voltage spikes in hESC-RPE cells, (2) documented rapid spontaneous changes in membrane conductance and resting potential, and (3) discovered that electrical excitation can spread to the neighboring cells in a syncytium comprised of the conventionally non-excitable cells.

### Spikes

Expression at significant levels of the classical voltage-activated Na^+^ conductances characterized by ultra-fast activation and inactivation is usually associated with generation of voltage spiking events, ranging in amplitude from small depolarizing waves to full-blown action potentials. Fast depolarizing voltage responses resembling action potentials have been previously described in the conventionally non-excitable cells such as astrocytes [20–22], rabbit pigmented ciliary epithelial cells [23], newt RPE [19], and neonatal rat RPE [16]. However, Botchkin and Matthews attributed the development of *I*_Na_ and the spiking to neuronal trans-differentiation of the cultured and actively proliferating RPE [16].

Previous studies documented expression of multiple types of voltage-activated Na^+^ and Ca^2+^ channels both in the cultured hESC-RPE and freshly dissected mouse RPE [4, 5]. *I*_Na_ can be large, raising a question about possible electrogenic function of Na^+^ channels in RPE. In comparison to the previous study [4], the patch-clamp experiments in this work were performed with series resistance compensated and under conditions as close as possible to those the cells were maintained in, i.e. at the temperature of ~34°C, in the physiological medium and using “all-ions” intracellular solution, so that *V*_rest_ was not compromised by added K^+^ channel blockers. We found that when *I*_Na_ was recovered from inactivation, depolarizing current pulses indeed evoked spikes, the amplitudes of which were mainly determined by the maximal amplitude of *I*_Na_ (Fig. 6A, D). Application of TTX strongly inhibited both *I*_Na_ and spikes (Fig. 4).

We did not detect any spikes triggered spontaneously without prior manipulations with membrane potential. Our results indicate that spontaneous spike generation is precluded by low absolute availability of *I*_Na_ at normal *V*_rest_ values and high *I*_Na_ activation threshold when *V*_rest_ was permissive. The availability of *I*_Na_ is a function of the maximal *I*_Na_ and the momentary inactivation state. The spikes could not be evoked at the group-average *V*_rest_ of −29 ± 18 mV because of near complete *I*_Na_ inactivation. However, in nine cells (Fig. 7F), *V*_rest_ transiently hyperpolarized to the levels of <−60 mV, at which a major fraction of *I*_Na_ could recover from inactivation (Fig. 2D–G and 7F–H). Then, if the maximal *I*_Na_ was high enough to yield a sufficiently large residual *I*_Na_ at the *V*_rest_, a depolarizing fluctuation of membrane potential could plausibly trigger a spike as we observed in the current-clamp experiments (Fig. 7). Although we routinely detected spontaneous fluctuations of *V*_rest_, a particularly large and rapid one would be required to reach the *I*_Na_ activation threshold potential at around −40 mV without a fatal loss of *I*_Na_ due to closed-state inactivation. Thus, a combination of several low-probability events is necessary to evoke a distinct spontaneous spike: a large *I*_Na_ expression level, several tens of ms long hyperpolarization to about −70 mV, and a rapid depolarization by about 30 mV (Fig. 7H).

Observations of *V*_rest_ fluctuations associated with changes in the putative depolarizing leak conductance (Fig. 8) suggest that addition of even a small repolarizing K^+^ conductance might be sufficient to permanently lower *V*_rest_. If hESC-RPE cells expressed sufficient quantities of conventional voltage-activated K^+^ channels [18] supporting sustained current with a low voltage activation threshold, group-average *V*_rest_ would be more hyperpolarized. This would not only increase availability of *I*_Na_ but also make the membrane faster, enabling rapid voltage transients. For instance, the cell in Fig. 7G, H is characterized by a very negative *V*_rest_ because the depolarizing leak conductance is presumably very small whereas *R*_m_ is high. Any transient depolarization can be filtered out by the very slow membrane, preventing rapid reaching of the *I*_Na_ activation threshold.

Interestingly, *V*_rest_ of the freshly isolated adult human and monkey RPE was on average −50 mV [12], being in similar range in mouse RPE monolayers [17], that is ~20 mV lower than in our hESC-RPE (differences in liquid junction potentials accounted for). Contributing to *V*_rest_, these cells also had prominent inward and delayed rectifier K^+^ channels [12] and expressed *I*_Na_, albeit only in subconfluent cultures [13] similarly to newt RPE [19]. Although the latter observation suggested that *I*_Na_ could be a feature of immature RPE [13], the recent findings demonstrate that Na^+^ channels in mature hESC- and mouse RPE monolayers are largely localized in the vicinity of the cell-cell junctions [4]. Dissociation of RPE destroys the intercellular contacts and drastically decreases whole-cell *I*_Na_ [4]. Therefore, the observed differences between the freshly isolated and the cultured RPE cells require caution in interpretation of results [4, 13, 16].

Although full-fledged spikes are one of the most spectacular excitation events in hESC-RPE, amplification of small transient depolarizations by the residual *I*_Na_ might be its main electrogenic function. Such depolarizations interspersed with hyperpolarizations are prevalent in our data (Figs. 7A and 9). As discussed previously, depolarizing *I*_Na_ could contribute by promoting the opening of slower L-type Ca^2+^ channels [37, 38]. Of Ca^2+^-dependent processes in the RPE, POS phagocytosis is a highly dynamic event requiring rapid actin remodeling for the phagocytic cup formation and phagosome ingestion (reviewed by [39]). As POS phagocytosis has been shown to depend on both L-type Ca^2+^ and Na^+^ channels [4, 5, 40, 41], depolarizing *I*_Na_ could facilitate L-type Ca^2+^ channel opening in the RPE participating in the complex interaction of molecular players involved in the Ca^2+^ regulation of phagocytosis [2].

Light response decreases the sub-retinal K^+^ concentration causing hyperpolarization [9] that together with large spontaneous fluctuations in the membrane resistance could trigger activation of *I*_Na_ in vivo and make RPE excitable, with excitation rapidly spreading in the monolayer. In addition, Na_V_1.8, one of the prominent subtypes present in hESC-RPE [4], has a comparatively depolarized half-inactivation potential of ~ −30 mV [42, 43]. If Na_V_1.8 contributes to any substantial level, its big portion would be available for activation at rest. Thus, taking into account the presence of several types of voltage-gated ion channels in the RPE, intrinsic properties of RPE membrane and its hyperpolarization in the transition from dark to light, the capacity for spiking makes sense and supports the role of RPE as much more versatile tissue than merely giving homeostatic support to the retina.

### Fluctuating membrane leak

Membrane conductance fluctuated in virtually all cells with current-clamp recordings. Similarly, in the voltage clamp experiments, changes in leak occurring in the course of seconds were also observed routinely. The finding of a large, spontaneously and rapidly fluctuating depolarizing conductance is unusual. However, the crucial question is whether the observed changes in *R*_m_ reflect genuine, ion channel-dependent changes in membrane conductance, or are due to seal instability. Although this problem cannot be solved decisively without additional pharmacological/genetic studies of ion channels that could mediate leak conductance(s), there are several arguments in support of the physiological leak hypothesis. Changes in leak conductance in the range from 10 to about 0.5 nS localized at the membrane-electrode interface in the whole-cell configuration usually cause irreversible seal disruption. A non-selective instrumental conductance caused by imperfect attachment of plasma membrane to the electrode glass would dissipate the local transmembrane ionic gradients in the vicinity of the membrane-electrode interface. This would immediately diminish the recorded voltage-activated ionic currents as is observed with bad seals, and lead to quick run-down. Our experience with other types of cells suggests that although seal quality can slowly improve over time, especially in the beginning of an experiment, and thus increase whole-cell resistance, the opposite leak-increasing changes quickly ruin the experiment. Continuous and rapid fluctuations of leak occurring repeatedly over the course of many minutes are inconsistent with the seal instability hypothesis.

Assuming that the fluctuating leak was physiological, what channels could mediate it? The leak current is overall depolarizing, indicating that K^+^ channels in the plasma membrane cannot dominate it above the K^+^ reversal potential of −90 mV. However, although membrane properties appeared to be passive both in high- and low-resistance states (Figs. 3B, E and 8F) suggesting that leak channels are not voltage-activated, changes in leak appear to be associated with strong changes in membrane potential (Figs. 7A, D and 8G–K). Thus, it is possible that when membrane is strongly hyperpolarized by a current pulse, voltage-dependent inward rectifier K^+^ channels [44, 45] open and provide rapid depolarization, contributing to the sharp depolarizing transients we observed (Fig. 9). However, these channels tend to close with depolarization (Fig. 1E). At membrane potentials more negative than the reversal potential for Cl^−^ (−39 mV in our experiments), efflux of Cl^−^ via various chloride channels [6, 8] could mediate a depolarizing leak current.

However, we argue that leak current fluctuations are probably caused by changes in gap junction conductance [46, 47]. Under physiological conditions, RPE cells face three external ionic milieus, two on the apical and basal surfaces and that/those of the neighboring cells. In our experiments, the trans-epithelial chemical gradient was disrupted but the intercellular ionic gradient probably exacerbated after the cytosol equilibrated with the patch pipette solution. RPE cells express Cx43-based gap junctions [11, 17, 48] that have voltage-dependent gating properties [47], so a strong depolarization or hyperpolarization of the clamped cell can change the transjunctional potential, leading to the opening or closing of gap junctions. This is consistent with the observation that sudden changes in leak were associated with strong changes in membrane potential during current-clamp experiments (Fig. 7A, D). Furthermore, recent studies reveal the presence of functional Cx43 hemichannels largely on the apical membrane of human and mouse RPE [11, 17]. Opening or closing of Cx43 hemichannels expressed in the plasma membrane could contribute to the changes in *R*_m_ observed in this work.

### Spread of electrical excitation in RPE

Delayed activation of sodium currents characterized by more positive reversal potential than that of the normal *I*_Na_ is often observed in neurons with elongated branches. The so-called escaping currents are caused by inadequate space clamp [49, 50]. In the small hexagonally shaped RPE cells, however, *I*_Na_ can escape not from a distal dendrite or axon but from the neighboring cells connected with open gap junctions.

Gap junctional coupling between cells in the RPE monolayer is likely to promote synchronization of various processes, and its role in eye development has been shown in the developing chick [48, 51]. While it is known that gap junctions in RPE allow the spread of electrical signals [17, 52], here we demonstrated evidence that electrical coupling could be strong enough to allow spread of *I*_Na_-mediated excitation waves to the neighbors of the patched cell (Fig. 10C, F).

We propose the following mechanism for the generation of the delayed inward currents observed in this study. When the patched cell is connected to its neighbors via open gap junctions, they introduce additional junctional resistance (*R*_GJ_) in series with the electrode series resistance (*R*_s_), so that charging of the neighboring cell is governed by the sum of two series resistances and its *R*_m_, and the higher the ratio of *R*_GJ_ +* R*_s_ to the second cell’s *R*_m_, the smaller the voltage drop on the membrane. Consequently, the neighboring cells can be clamped via gap junctions but the eventual membrane voltage and the speed of its change will depend on *R*_GJ_ +* R*_s_. When *R*_GJ_ is relatively small, the clamping of the second cell, while accomplished with probably tens of mV voltage errors, could still yield enough *I*_Na_ to be detected across the junction. Conversely, when *R*_GJ_ is large, the other cell’s voltage cannot be commanded to a degree sufficient to either recover *I*_Na_ or reach its activation threshold. Thus, multiple delayed currents probably arise in different neighboring cells with dissimilar but permissive *R*_GJ_ values, and if *R*_GJ_ values of two or more connections are about the same, their delayed currents would merge. Similar but comparatively slow processes can occur during current injections in the current-clamp experiments.

It is even possible that if *R*_GJ_ is very low, providing large conductance, the delayed current can merge with the primary *I*_Na_. If *R*_GJ_ can indeed fluctuate dynamically then this might explain the broadening of *I*_Na_ in Fig. 10H right sub-panel compared to the left sub-panel. However, such cells would still be characterized by slow settling of the capacitance transient in the voltage clamp indicative of clamping across the junctions.

It appears that in the absolute majority of RPE cells the *R*_GJ_ was too high to enable delayed inward currents. This is supported by our experiments with Cx43 inhibitor 18α-GA, which resulted in a significant but comparatively small effect on membrane capacitance (Fig. 11F). While the cause of observed variability in gap junctional conductance is not clear, in addition to the transjunctional potential, gap junctions are regulated by many mechanisms, including internal Ca^2+^ concentration, elevation of which can rapidly and completely close the gap junction channels [35, 53, 54]. It was reported that increase of the cytosolic Ca^2+^ concentration after application of the calcium ionophore ionomycin resulted in a reduction of Cx43 gap junction conductance by a chemical gating mechanism [53]. In RPE, calcium can enter the cell from the extracellular space via TRP and L-type Ca^2+^ channels, from the endoplasmic reticulum via IP_3_R channels, and from the neighboring cells via gap junctions [6, 51], and these sources might manifest dissimilarly in different cells.

It is possible that in the cells where delayed inward currents were observed, L-type Ca^2+^ channels could be the main source of Ca^2+^ influx. When voltage in such cells is held below the L-type Ca^2+^ channel activation threshold, either by clamping membrane potential to −80 mV in the voltage clamp, or by injecting a hyperpolarizing current in the current clamp, the influx of Ca^2+^ would be minimized and the gap junction blockade relieved. However, when the cell is depolarized during the test pulses, inward Ca^2+^ current would resume, partly blocking the gap junction channels. Then, as nifedipine blocked the L-type Ca^2+^ channels, Ca^2+^ influx stopped even during the depolarizations, thus maximizing the gap junction conductance, and increasing the delayed inward current amplitude and speeding up its onset (Fig. 11). Alternatively, cells characterized by the delayed inward currents could express more gap junction channels so that the same fraction of gap junction conductance as in the “normal” cells would produce sufficiently low *R*_GJ_ to elicit *I*_Na_. Finally, it is worth keeping in mind that Cx43 gap junctions are suggested to be involved in ephaptic coupling, e.g., in vertebrate retina [55] and heart [56], strengthening the spread of depolarization to the nearby cells. We have previously shown that in RPE, both gap junctions and voltage-gated Na^+^ channels localize to the tight junctions between the cells [4, 17] generating conditions where neighboring cell membranes containing these channels are closely associated. This could support ephaptic coupling eliciting an intriguing aspect for future studies.

## Conclusions

The present work reveals how non-neuronal RPE cells residing in vivo in the immediate interactive contact with neuronal retina have active intrinsic properties. Remarkably, these cells can change the properties not only as response to the relatively slow changes in the ionic milieu associated with illumination-dependent changes in photoreceptor functioning, but also rapidly, on the scale of seconds, influence the electrophysiological states of the neighboring cells. Our studies demonstrate that in RPE, currents through Na^+^ channels mediate voltage spikes that can spread laterally in the monolayer through gap junctions. In addition, membrane resistance in the RPE shows immense and spontaneous fluctuations causing correlated alterations in the membrane potential. These properties enable mutual and previously uncovered interaction between RPE and the neural retina yet requiring further investigation with undisturbed physical interaction between the two tissues.

## Methods

### Cell culture

Two previously derived hESC lines Regea08/017 and Regea11/013 were used in this study. These lines have been established from the blastocyst-stage human embryos using mechanical derivation methods [57]. The hESC lines were cultured and differentiated into RPE as previously described [58, 59] followed by cryopreservation [28, 60]. The cells were plated on hanging cell culture inserts (polyethylene terephthalate membrane with 1.0 μm pore size, Sarstedt, Inc., Newton, NC, USA) coated with Biolaminin LN 521 (1.8 μg/cm^2^) and Collagen IV (10 μg/cm^2^) with a density of 5.5 × 10^5^ cells/cm^2^. Inserts were placed in 24-well plates (Corning Inc., Corning, NY, USA) and cell monolayers nourished from both the apical and basolateral sides. For our experiments, the cells were cultured at +37 °C in 5% CO_2_ in Knock-Out Dulbecco’s modified Eagle’s medium (KO-DMEM) containing 15% Knock-Out serum replacement (KO-SR), 1% Glutamax, 0.2% 2-mercaptoethanol (all from Thermo Fisher Scientific, Waltham, MA, USA), 1% non-essential amino acids (NEAA), and 50 U/mL penicillin/streptomycin (both from Lonza Group, Basel, Switzerland). The medium was replenished three times a week.

### Patch-clamp recordings

Cells cultured for 4–26 weeks from several independent differentiation batches were used. Current and voltage recordings were obtained from hESC-RPE monolayers using the standard patch-clamp technique in the whole-cell configuration. Patch-clamp pipettes were pulled from borosilicate glass capillaries (0.86 mm I.D. 1.5 mm O.D., Science Products GmbH, Hofheim, Germany and Harvard apparatus, Holliston, MA, USA) using a filament-based puller P-1000 (Sutter Instrument, Novato, CA, USA). Pipettes were filled with an internal solution containing (in mM): 83 K-gluconate, 25 KCl, 5.5 EGTA, 0.5 CaCl_2_, 4 ATP-Mg, 0.1 GTP-Na, 10 HEPES, and 5 NaCl (pH adjusted to 7.2 with KOH and osmolarity to 290 mOsm with sucrose). The final electrode resistance was between 4 and 7 MΩ. In some experiments (Fig. 11F–K and Additional file 1. Fig. S1), K-gluconate (83 mM) and KCl (25 mM) in the pipette solution were replaced with CsCH_3_SO_3_ (83 mM) and CsCl (25 mM).

RPE monolayers were continuously superfused with Ames’ solution (Sigma-Aldrich, St. Louis, MO, USA) supplemented with 10 mM HEPES and 10 mM NaCl (pH adjusted to 7.4 with NaOH and osmolarity to 315 mOsm with sucrose).

Extracellular solution was heated to 33–35 °C with the inline heater SH-27B controlled with a TC-324B unit (Warner Instruments, Hamden, CT, USA). In some experiments, nifedipine or 18α-glycyrrhetinic acid (both 10 μM, Sigma-Aldrich, St. Louis, MO, USA), or TTX citrate (10 μM, Abcam, Cambridge, UK) was added to the bath solutions.

All recordings were made using Axopatch 200B patch-clamp amplifier connected to Digidata 1440A converter (Molecular Devices, San Jose, CA, USA). Currents were recorded at the sampling frequency of 250 kHz and low-pass filtered at 2 kHz. Holding potential was −70 mV in all voltage clamp experiments. Resting membrane potential (*V*_rest_) was determined from the current clamp in the absence of current injection. Only recordings with series resistance of ≤ 25 MΩ were used. Liquid junction potential calculated according to the Nernst-Planck equation was +13 mV. Data presented in the text and figures were not compensated for the liquid junction potential. Capacitance compensation was used to full extent. In the majority of cells, series resistance was compensated by the built-in circuit of the amplifier (20–80% correction).

Equilibrium potentials calculated from the patch pipette and bath solutions concentrations were −90.1 mV for *E*_K_, 85.8 mV for *E*_Na_, 221.7 mV for *E*_Ca_, and −39.3 mV for *E*_Cl_.

### Data analysis and statistics

The character of data distribution was measured using the Shapiro-Wilk normality test. Data in the samples that generally did not pass the normality test are presented in the text as medians with 1st:3rd interquartile ranges. In pairwise comparisons, such data were statistically compared using Mann–Whitney *U*-test (MWUT). All normally distributed cumulative data were presented as means ± standard deviation (s.d.). Significance testing was performed using the *t*-test as indicated. Pearson’s correlation coefficient (*r*) was used for the analyses of correlations. Throughout the text, (*n*) denotes the group size.

## Supplementary Information

**Additional file 1:** **Fig. S1. **Kinetics of *I*_Na_ recorded using cesium-based intracellular solution

**Additional file 2:** **Table S1. **Datasets

## Data Availability

All data generated or analyzed during this study are included in this published article, its supplementary information files (Additional file 1: Fig. S1, Additional file 2: Table S1), and Figshare data repository [61]. Where *n* < 6, the individual data values are provided in additional files (Additional file 2: Table S1) and cited in the figure legends.

## Abbreviations

*C*_m_: Membrane capacitance
*g*: Conductance
hESC-RPE: Human embryonic stem cell-derived retinal pigment epithelium
*I*_C_: Capacitive current
*I*_IR_: Sustained hyperpolarization-activated inward current
*I*_Na_: Voltage-activated Na^+^ current
*I*_K_: Voltage-activated K^+^ current
*I*_A_: Transient outward voltage-activated K^+^ current
*I*_DR_: Sustained voltage-activated K^+^ current
POS: Photoreceptor outer segments
*R*_m_: Membrane resistance
*R*_GJ_: Junctional resistance
*R*_s_: Series resistance
SSI: Steady-state inactivation
TTX: Tetrodotoxin
*V*_rest_: Resting potential
*τ*_m_: Membrane time constant
*τ*_rec_: Recovery time constant
18α-GA: 18α-glycyrrhetinic acid
