# Supplementary material for: The retinal pigment epithelium displays electrical excitability and lateral signal spreading
Source: BMC Biol. 2023 Apr 17;21:84. doi: 10.1186/s12915-023-01559-5 (PMC10111697; doi:10.1186/s12915-023-01559-5)
Supplement: Supplementary file 1 — Additional file 1: Fig. S1. Kinetics of INa recorded using cesium-based intracellular solution [file 12915_2023_1559_MOESM1_ESM.pdf]

Additional file 1: Figure S1.

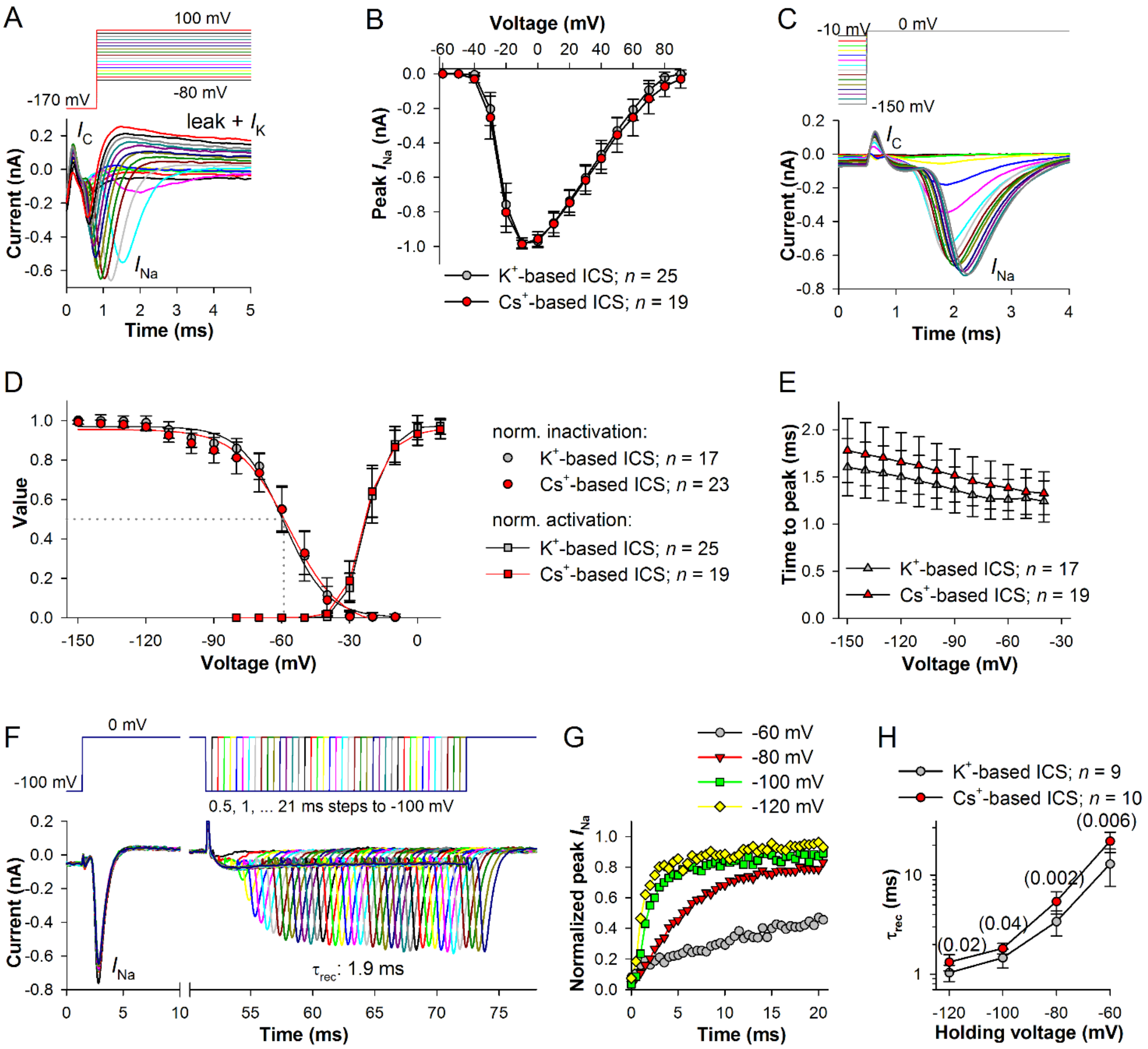

Additional file 1: Figure S1. Kinetics of I_{Na} recorded using cesium-based intracellular solution. **A** Typical I_{Na} evoked by depolarizing pulses from -80 to +100 mV in 10 mV increments after holding the cell at -170 mV for 50 ms; I_C, residual capacitive current; I_K, the residual voltage-activated K^+ current. In these experiments K-gluconate (83 mM) and KCl (25 mM) in intracellular solution (ICS) were

replaced with $CsCH_3SO_3$ (83 mM) and CsCl (25 mM). Cs^+-based sample also contains several cells recorded in extracellular solution supplemented with 10 µM nifedipine. **B** Comparison of average *I*-*V* relationships for I_{Na} recorded using K^+-based (25 cells) and Cs^+-based (19 cells) solutions; here and elsewhere error bars are s.d. (Additional file 2: Table S1). **C** Example of a SSI experiment with Cs^+-based solution. SSI was studied by evoking I_{Na} at 0 mV after a 50 ms conditioning pre-pulses from -150 to -10 mV. **D** Voltage-dependencies of the normalized peak I_{Na} from SSI experiments and the normalized peak I_{Na} conductance. SSI data were divided by I_{Na} after the pre-pulse to -150 mV; average values were fitted with a sigmoidal equation. Conductance at each membrane potential *V* was calculated using peak I_{Na} from *I*-*V* relations as in B and equation $g_{Na}(V) = I_{Na}/(V - E_{rev})$, where E_{Na} = 85.8 mV (Additional file 2: Table S1). **E** Voltage-dependencies of the peak I_{Na} time to peak from SSI experiments for two intracellular solutions (Additional file 2: Table S1). **F** Recovery of I_{Na} from inactivation with Cs^+-based internal solution. I_{Na} was evoked and inactivated by a 50 ms pre-pulse to 0 mV and then hyperpolarizing pulses of increasing duration ranging from 0.5 to 21.0 ms were applied. In this experiment, the test holding potential was -100 mV. **G** By plotting peak I_{Na} during recovery from inactivation against the hyperpolarizing pulse duration and then fitting the data with a first-order exponential rise-to-maximum equation, recovery time constants (τ_{rec}) can be obtained; examples are from the same cell as in F. **H** Comparison of average voltage-dependence of the recovery time constants from inactivation for two intracellular solutions; unpaired *t*-test was used for the statistical comparison (Additional file 2: Table S1).

Kinetics of I_{Na} were compared between RPE cells recorded using K^+-based and Cs^+-based internal solutions. *I*-*V* relationships, voltage-dependencies of activation and inactivation were identical (Additional file 1: Fig. S1 A-D). I_{Na} reached peak more slowly in Cs^+-based internal solution experiments

compared to K^+-based solution. However, the differences were not statistically significant (Additional file 1: Fig. S1 E, unpaired *t*-test). This finding is consistent with results of a previous study [33].

Recovery time constants of I_{Na} from inactivation were statistically significantly slower when Cs^+-based internal solution was used (Additional file 1: Fig. S1 F-H, unpaired *t*-test).

Adding 10 µM nifedipine to the extracellular solution did not affect kinetics of I_{Na}; therefore, Cs^+-based sample also contains several cells recorded in extracellular solution supplemented with 10 µM nifedipine.
